# Incidental Anomalous Left Coronary Artery in a Transplanted Heart

**DOI:** 10.1155/2019/2715896

**Published:** 2019-12-26

**Authors:** Sri Harsha Patlolla, Saraschandra Vallabhajosyula, Malcolm R. Bell

**Affiliations:** Department of Cardiovascular Medicine, Mayo Clinic, Rochester, Minnesota, USA

## Abstract

Anomalous coronary artery is an uncommon congenital cardiac anomaly that is often detected incidentally on coronary angiography. It has rarely been reported in the donor heart of patients who have undergone cardiac transplantation. Here, we report a case of a 72-year-old patient who received a second heart transplant and has been identified to have an anomalous left main coronary artery originating from the right coronary sinus on postoperative coronary angiography.

## 1. Introduction

Anomalous coronary artery arising from the opposite sinus has been reported in less than 2% of the population [1], and the reported frequency of a left main coronary artery arising from the right coronary sinus is less than 0.2% [2, 3]. The anomalous origin of coronary arteries has been infrequently noted in patients with prior cardiac transplantation. Herein, we report a case of an anomalous left coronary artery in a patient with a prior cardiac transplantation.

## 2. Case Report

A 72-year-old male presented to the Mayo Clinic Cardiac Catheterization Laboratory for routine posttransplant surveillance coronary angiography after undergoing a second orthotopic heart transplantation at another institution four months ago. He received his first heart transplant 30 years ago for dilated cardiomyopathy which was complicated by progressive cardiac allograft vasculopathy. In 2019, he was listed and received a second cardiac transplantation at an outside medical center. The early postoperative course was uneventful, and he was on a stable immunosuppressive regimen with prednisone, tacrolimus, and mycophenolate mofetil. A postoperative coronary angiography was attempted four weeks following the transplantation, but the left coronary artery could not be selectively engaged due to the reported inferior origin of the left main artery with likely posterior rotation. He reported being told by his physicians that they “were unable to find his heart arteries.” He subsequently transferred his posttransplant care to the Mayo Clinic. As a part of the routine postoperative surveillance, he was scheduled for a coronary angiogram, right heart catheterization, and endomyocardial biopsy.

**Figure 1:**
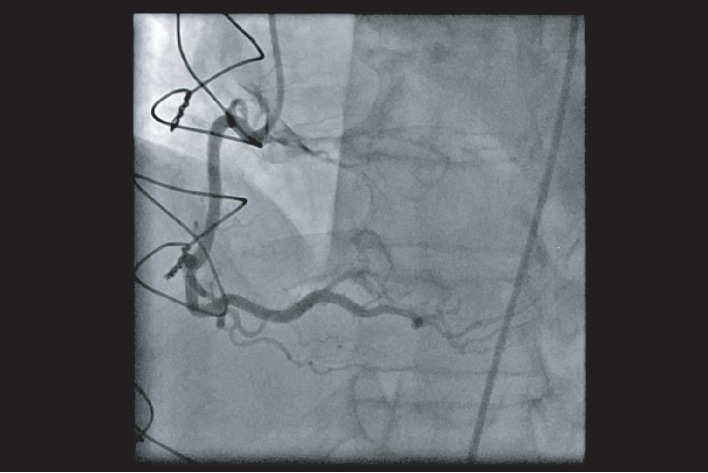
Right coronary artery arising from the right coronary sinus. Left anterior oblique cranial view showing a dominant right coronary artery arising from the right coronary sinus.

**Figure 2:**
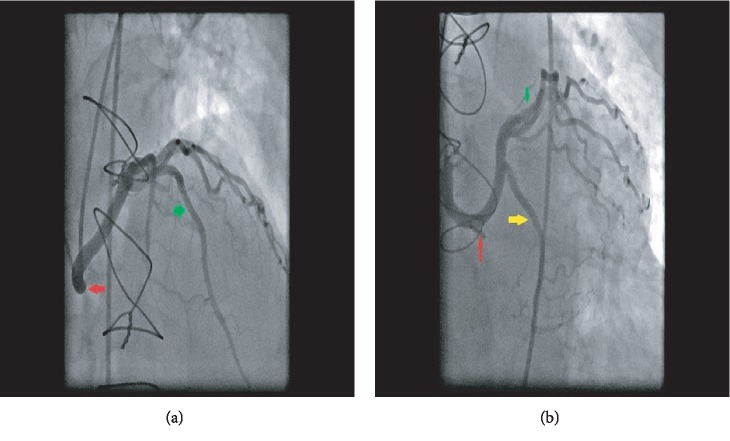
Anomalous left coronary artery arising from the right coronary sinus. Right anterior oblique cranial view (a) and left anterior oblique caudal view (b) showing an anomalous left coronary artery arising from the right coronary sinus with a long left main artery (red arrows) that bifurcates into a left anterior descending (green arrows) and left circumflex coronary artery (yellow arrow).

**Figure 3:**
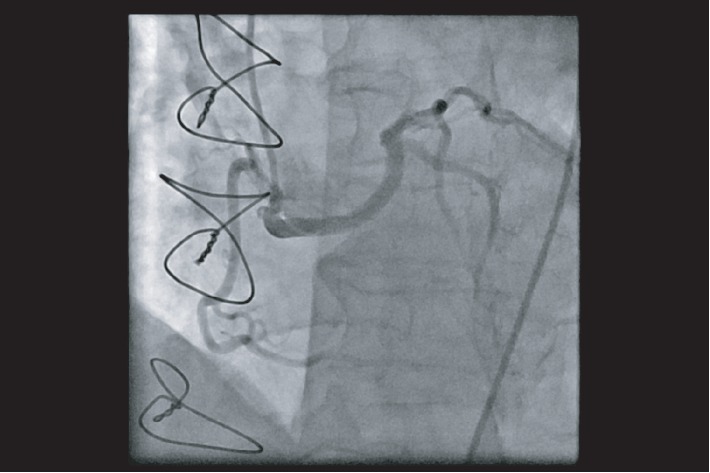
Right coronary artery and anomalous left coronary artery arising from the right coronary sinus. Left anterior oblique view showing a semiselective injection of the right coronary sinus with two discrete origins for the right coronary artery and anomalous left coronary artery.

The review of the earlier angiogram had raised the suspicion of an anomalous left coronary artery. During coronary angiography, the right coronary artery was engaged without difficulty using a 6Fr Williams right catheter and looked normal in appearance (Figure 1 and Online [Supplementary-material supplementary-material-1]); the left coronary artery was also seen arising posterior-inferiorly adjacent to the right coronary ostium. Selective injection from this position using a 6Fr multipurpose catheter revealed an anomalous left coronary artery arising from the right coronary sinus (Figures 2(a), 2(b), and 3 and Online Videos [Supplementary-material supplementary-material-1], [Supplementary-material supplementary-material-1], and [Supplementary-material supplementary-material-1]). The long left main artery followed a retroaortic course before bifurcating into the left anterior descending and left circumflex arteries which otherwise appeared normal in appearance. We had discussed obtaining a dedicated coronary computerized tomographic scan to delineate his coronary anatomy; however, given his advanced renal disease, this was deferred after discussion with colleagues in heart failure and cardiac transplantation. Given his asymptomatic status, he was managed conservatively with routine posttransplant surveillance.

## 3. Discussion

Though coronary anomalies are mostly benign, in some cases, they are known to cause ischemic changes that can precipitate myocardial infarction or sudden cardiac death [4]. A nationwide study reported coronary anomalies originating from the opposite sinus as the second most common cause of sudden cardiac death in young athletes [5]. Such clinically significant events are mostly dependent on the course taken by the anomalous left coronary artery when arising from the opposite sinus [6]. An interarterial course between the aorta and pulmonary artery is often associated with sudden cardiac death. Retroaortic, anterior, and transseptal courses of the anomalous left coronary artery have less frequently been associated with adverse events [4, 7]. Although a retroaortic course is generally considered to have a minimal risk of sudden cardiac death, there have been reports of its association with sudden death and ischemic events [2, 8, 9]. Our patient did not have any clinical symptoms necessitating intervention.

Coronary artery anomalies are a rare finding especially in heart transplant recipients and are mostly benign. They are usually identified on postoperative coronary angiograms [1, 4]. As most heart donors are young patients who are unlikely to have any preexisting indications for angiography, it is difficult to detect such anomalies prior to transplant. However, if coronary anomalies are suspected, it is important to consider the future risk of complications before rejecting the donor heart. A careful evaluation of the coronary ostia on transthoracic echocardiography should be able to identify most coronary anomalies. Computerized tomographic coronary angiography and magnetic resonance coronary angiography have been suggested as a class IIA/B recommendation for the evaluation of anomalous coronary arteries by the American Heart Association Committee on Cardiovascular Imaging [10]. Our patient had stage III chronic kidney disease, and thus, follow-up confirmatory computerized tomographic coronary angiography was deferred. Currently, there is limited evidence on the role of cross-sectional imaging for coronary anomalies in donor hearts. Coronary anomalies in native hearts are prone to spontaneous coronary spasm in 1/3 of the cases [11]. However, this may not be perceived in a donor heart but spasm may be considered, along with appropriate testing, if there is suspicion of angina and/or ischemia [12]. Coronary anomalies can be managed either medically or surgically depending on the presentation. In general, an individualized approach based on the patient's age group, clinical presentation, and consideration of the risk of sudden cardiac death is advocated [1, 13–17].

## Supplementary Materials

Supplementary Materials Video 1: right coronary artery arising from the right coronary sinus. Video 2: anomalous left coronary artery arising from the right coronary sinus. Video 3: right coronary artery and anomalous left coronary artery arising from the right coronary sinus.
